# Mast Cells Present Protrusions into Blood Vessels upon Tracheal Allergen Challenge in Mice

**DOI:** 10.1371/journal.pone.0118513

**Published:** 2015-03-19

**Authors:** Oishee Bose, Peter Baluk, Mark R. Looney, Laurence E. Cheng, Donald M. McDonald, George H. Caughey, Matthew F. Krummel

**Affiliations:** 1 Department of Pathology, University of California San Francisco, San Francisco, California, United States of America; 2 Department of Anatomy, University of California San Francisco, San Francisco, California, United States of America; 3 Department of Laboratory Medicine, University of California San Francisco, San Francisco, California, United States of America; 4 Department of Pediatrics, University of California San Francisco, San Francisco, California, United States of America; 5 Department of Medicine, University of California San Francisco, San Francisco, California, United States of America; 6 Cardiovascular Research Institute, University of California San Francisco, San Francisco, California, United States of America; 7 Helen Diller Family Comprehensive Cancer Center, University of California San Francisco, and Veterans Affairs Medical Center, San Francisco, California, United States of America; French National Centre for Scientific Research, FRANCE

## Abstract

Mast cells (MC) and myeloid dendritic cells (DC) act proximally in detecting and processing antigens and immune insults. We sought to understand their comparative dynamic behavior with respect to the airway epithelium in the steady state and in response to an allergic stimulus in mouse trachea. We devised methods to label MC in living trachea and to demonstrate that MC and DC occupy distinct layers of the tracheal mucosa, with DC being closer to the lumen. DC numbers doubled after allergen challenge, but MC numbers remained stable. MC and DC migrated minimally in either steady state or allergen-challenge conditions, and their interactions with one another appeared to be stochastic and relatively infrequent. While DC, unlike MC, exhibited probing behaviors involving dendrites, these projections did not cross the epithelium into the airway lumen. MC typically were located too far from the epithelial surface to contact the tracheal lumen. However, MC had protrusions toward and into blood vessels, likely to load with IgE. Thus, DC and MC occupy distinct niches and engage in sessile surveillance in the mouse trachea. Little or no access of these cell types to the airway lumen suggests that trans-epithelial transport of proteins in the steady state would be required for them to access luminal antigens.

## Introduction

Live imaging of immune cells within their native tissue environments has revealed insights about how the immune system surveys and protects these tissues [1]. Multiphoton imaging, with its ability to penetrate deeper into tissue with less phototoxicity than with conventional imaging modalities, provides special benefit, because behaviors can be assessed within larger volumes, including some entire organs. For lymph nodes, tumors, skin, and lung, this imaging can be achieved in fully intravital surgical preparations or in organ explants using oxygenated media with or without partial sectioning. While progress has being made in imaging using multiphoton approaches to explore immune reactivity in the lung itself [2], the upper respiratory tract and trachea have been explored less in this regard.

Responses to inhaled materials can be achieved through a variety of immune cells, including mast cells (MC), which can detect antigen via IgE antibodies bound to surface Fc receptors and then degranulate to liberate vasoactive amines, lipid mediators, cytokines and proteases. In the skin, Cheng et al. used labeled IgE to reveal that MC make cellular projections into blood vessels, from which MC load IgE onto the cell surface [3]. Less is known of how MC behave in airway tissues and whether they make similar projections to sample contents of airway lumen.

Another critical cell population in the steady state respiratory tract is represented by myeloid cells such as dendritic cells (DC), which are characterized morphologically by dendritic protrusions and by the ability to phagocytose and process material for presentation to T cells. In mice bearing the CD11cYFP allele, DC and many macrophage populations have a permanent fluorescent label, permitting detection without adding dyes [4]. Using CD11cYFP alleles, DC have been shown to extend dendrites from the tissue into the lumen to sample the contents of lung alveoli [5] and proximal jejunum [6]. It has been speculated that DC in the trachea make similar projections into the airway lumen [7]. We hypothesized that DC might also serve to load MC with antigen via cell-cell contacts.

To explore these possibilities, we used multiphoton imaging and IgE labeling to compare dynamics of MC and DC populations in the trachea under baseline and allergic conditions. Our findings suggest that there is little or no sampling of tracheal luminal contents by mucosal DC and MC, and that both cell types are abundant in the trachea but have little opportunity to interact in the epithelium or lumen. Therefore, in the absence of tissue remodeling, these two populations of sentinel cells in the trachea likely encounter antigen that has crossed the epithelium.

## Results

### Distribution of mast cells in lung and trachea

**Fig 1:**
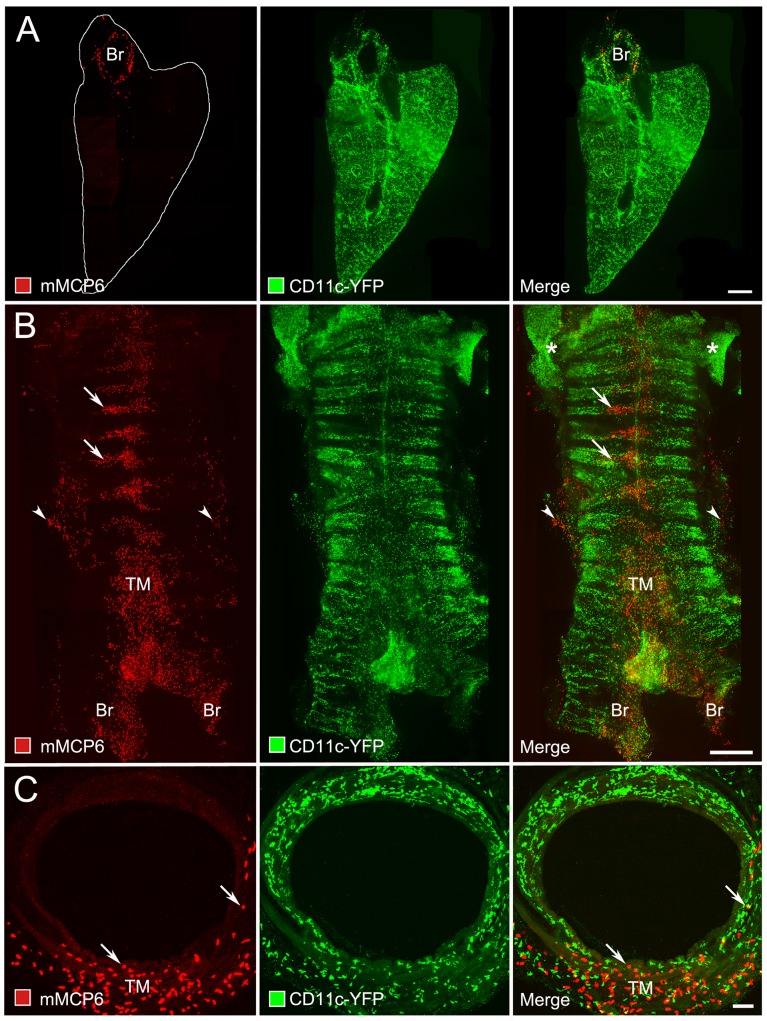
Distribution of MC in mouse airways. Lung and trachea of a pathogen-free mouse was stained to show MC (mouse mast cell protease 6, mMCP6, red) and dendritic cells (CD11c-YFP, green) imaged by fluorescence (A, B) or confocal microscopy (C). (**A**) Section (thickness, ~ 100 μm) of left lung without prior allergen challenge cut in a transverse plane near the hilum. Most MC are around the main stem bronchus (Br, left panel), whereas DC are more widely distributed and more abundant (middle and right panels). Bar: 1 mm. (**B**) Whole mount of trachea and proximal bronchi (Br) showing abundant MC between cartilage rings (horizontal black bands) and near the trachealis muscle (TM) of the posterior membrane, and some in the overlying adventitia (left panel, arrowheads). DC are more widely distributed and abundant in the trachea and in the thyroid gland (*) (middle and right panels). Bar: 1 mm. (**C**) Cross-section (thickness, ~ 100 μm) of trachea showing abundant MC in the adventitia over the trachealis muscle (left panel). DC are more abundant than MC and distributed around the entire tracheal circumference (middle and right panels). Bar: 100 μm.

As our ultimate goal was to understand MC and DC movement and interactions in the living trachea, we first used conventional immunohistochemistry to determine the distribution of MC in the respiratory tract of C57Bl/6 mice. We examined paraformaldehyde-fixed sections and whole mounts of lungs and tracheas of pathogen-free mice under baseline conditions, without prior allergen challenge. We labeled MC with antibodies against mouse MC-specific protease-6 (mMCP6) and identified DC by the yellow fluorescent protein (YFP) allele in *CD11c*-YFP mice. In the lung, most MC were restricted to the region of the hilum and main stem bronchus; few MC were found in the lung parenchyma (**Fig. 1A**). In comparison, DC were much more abundant and widely distributed in the lung. MC were abundant in the trachea, but were most numerous in the trachealis muscle (**Fig. 1B, C**). Fewer MC were in the mucosa between cartilage rings or near the tracheal epithelium (**Fig. 1B, C**). DC were more abundant and widespread than MC in the trachea. Although the populations overlapped, DC were generally closer than MC to the epithelium, except near the trachealis muscle (**Fig. 1C**).

### Approach for labeling and imaging of MC in living tracheas

**Fig 2:**
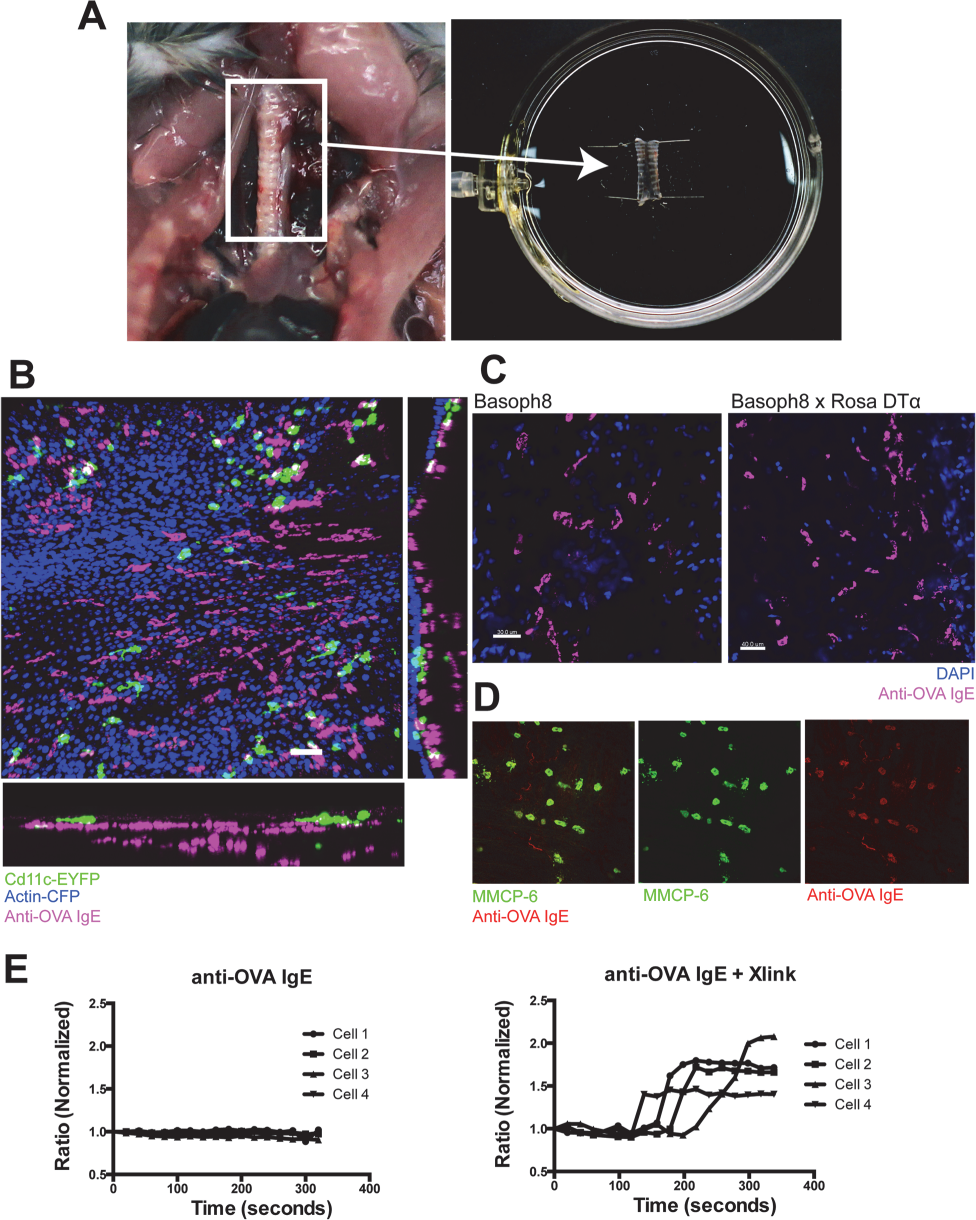
Mast cell staining and imaging methods and validation. (**A**) Method for excising and mounting trachea for imaging. (**B**) Baseline anti-OVA Alexa 647-IgE MC staining in the trachea of a CD11c-EYFP x Actin-CFP mouse. (**C**) Anti-OVA Alexa 647-IgE MC staining in trachea of the Basoph8 reporter strain and the basophil deficient Basoph8 × Rosa-DTα. (**D**) Co-localization of mMCP6 and anti-OVA Alexa 647-IgE in fixed, permeabilized tracheal whole mount. The background red staining of non-MC was an artifact introduced by the fixation necessary for mMCP6 staining and was not present in unfixed trachea. (**E**) Normalized ratiometric measurement of cytosolic Ca^2+^ flux in FURA-2AM-loaded, IgE receptor-positive RBL-2H3 cells. Cells were incubated with anti-OVA Alexa 647-IgE, then Ca^2+^ flux was measured with or without addition of anti-Mouse IgE (cross-linker).

Based on the distribution of mMCP6-positive MC in the trachea, we explored multiple methods for labeling MC in situ. For imaging MC in the trachea, we modified the slice method previously validated for lung [5,8]. Briefly, the trachea was excised and slit lengthwise to create a flat sheet of tissue and expose the lumen (**Fig. 2A**). We tested multiple MC protease promoter-GFP and ckit-GFP reporter mice but found that they did not effectively highlight MC in the mouse respiratory tract (data not shown). Adoptive transfer of GFP-expressing bone marrow-derived, cultured MC to MC-deficient *Kit* ^*W-sh*^ */Kit* ^*W-Sh*^ mice did not achieve high-level reconstitution in the proximal airway. We therefore modified the approach of Cheng et al. [3] to take advantage of a monoclonal mouse IgE antibody with specificity for ovalbumin (anti-OVA IgE) [9]. By incubating tracheal preparations with Alexa647-conjugates of anti-OVA IgE, we obtained strong surface labeling of cells with sizes and locations matching those seen after immunohistochemical staining (**Fig. 2B**). Depending on tissue microenvironment, many tracheal MC had elongated morphologies.

Given that basophils and MC both express Fc receptors for IgE, we sought to test more vigorously whether the cells we highlighted were indeed MC. **Fig. 2C** shows staining of trachea in mice that either had basophils (Basoph8 Cre allele alone) or lacked them (Basoph8 (Cre) x Rosa-DTα) [10]. This demonstrates persistence of IgE-binding cells in basophil-depleted tissue. By contrast, no IgE-labeled cells were found in the lung of basophil-depleted mice (data not shown). When immunostaining for mMCP6 was combined with IgE labeling, the two colocalized in most cells (**Fig. 2D**), indicating that most or all IgE-labeled cells in the trachea were in fact MC. Although IgE binding is thought not to initiate MC signaling, and thus Alexa 647-IgE staining should not alter cellular function, cross-linked IgE can induce signaling in MC. To determine whether our Alexa 647-IgE antibody contained multimeric forms sufficient to induce signaling, we loaded RBL-2H3 cells with Alexa 647-IgE conjugate and assessed initiation of an intracellular signal by calcium imaging (**Fig. 2E**). Incubation of cells with Alexa 647-IgE alone did not induce a calcium signal. However, when cell-bound IgE was cross-linked with anti-IgE secondary antibody, robust calcium transients were observed. This indicates that Alexa 647-IgE itself is not likely to initiate signaling and change cell behavior in living tracheas.

### Differential distribution and motility of tracheal MC and DC after allergen challenge

**Fig 3:**
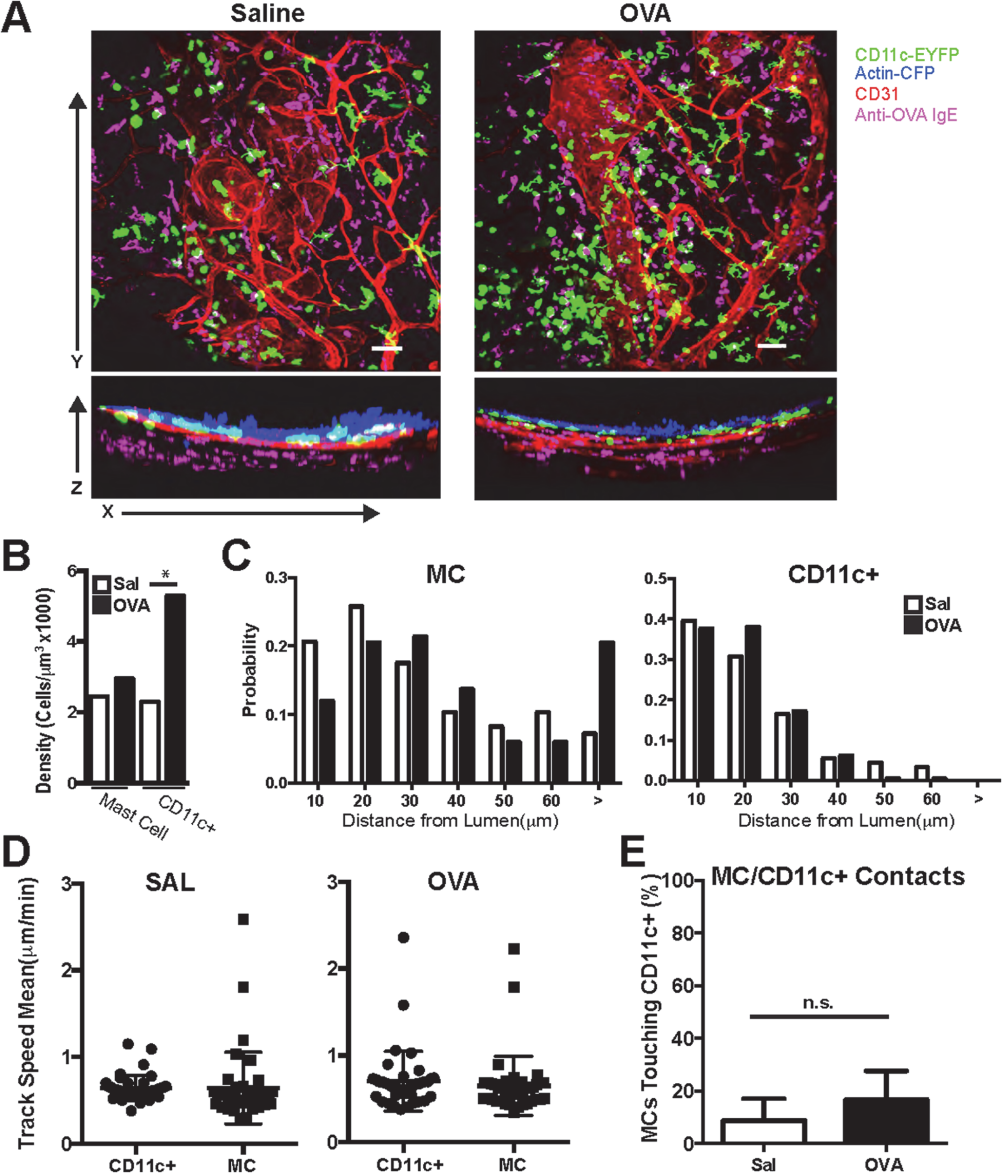
Spatiotemporal interactions of anti-OVA IgE Alexa 647 MC and DC in PBS- and OVA-challenged tracheas. (**A**) Spatial distribution of anti-OVA IgE Alexa 647 MC and CD11cYFP DC in saline (SAL)-challenged or OVA-challenged trachea. Tracheas were analyzed one day after the third OVA challenge (i.e., on day 24 of the OVA challenge regimen [5]). Side views show distribution in relation to the lumen. Scale bar = 50 μm (see also **S1 Movie**) (**B**). Density of DC and MC was determined by counting each cell type in images and measuring the complete volume of tissue and expressing data as cells/volume. (**C**) Quantified distribution of anti-OVA IgE Alexa 647 MC and CD11cYFP DC relative to the mucosal edge of tracheas after PBS or OVA challenge. Total cells per region were counted in at least 4 tracheas and expressed as a probability. (**D**) Mean track speed of anti-OVA IgE Alexa 647-stained mast cells and Cd11cYFP dendritic cells in PBS- and OVA-challenged tracheas. (**E**) Percent of MCs that had a cell-cell contact when viewed from all angles (see also **S2 Movie**).

We next sought to determine the dynamic behavior of MC and DC in living tracheal preparations. To this end, we used the approach just described and introduced antibodies to CD31 to highlight blood vessels of the trachea and performed live-imaging, collecting large volumes at 5-μm step size every 20 seconds to 1 minute for 20 minutes up to 1 hour. As shown in **Fig. 3A** and quantified in **Fig. 3B**, after allergen challenge, DC were more numerous but the number of MC was little changed. In addition, Z-projections (**Fig. 3A**) demonstrated and measurements confirmed (**Fig. 3C**) that MC were distributed broadly through the ~60–100 μm thickness of the tracheal wall. In contrast, ~80% of DC were located within 20 μm of the luminal surface of the epithelium. Across multiple sections quantified (**Fig. 3C**), there was no significant difference between the spatial distribution of either cell type in the trachea of saline- and ovalbumin-challenged mice.

We then viewed the time-lapse data to follow the movement of DC and MC. DC and MC moved at a rate of less than 1 μm per minute (**Fig. 3D, S1 Movie**), suggesting that neither cell type engaged in active ‘search’ of the type seen, for example, in T cells surveying lymph nodes [11]. The comparatively sessile behavior of DC and MC is more consistent with cells whose sentinel strategy is to remain in a relatively fixed location and await delivery of material to them, rather than undertaking active migration to survey new material. In addition, analysis of 3D image sequences over time revealed little direct contact between DC and MC. Only 10–15% of MC made contact with DC. While the mean number of MC making contact with DC was marginally higher when allergens were present, the difference was not statistically significant (**Fig. 3E** and **S2 Movie**) and likely resulted from higher overall DC densities in allergy-challenged tracheas (see Fig. 3B).

### Probing behaviors of DC versus MC

**Fig 4:**
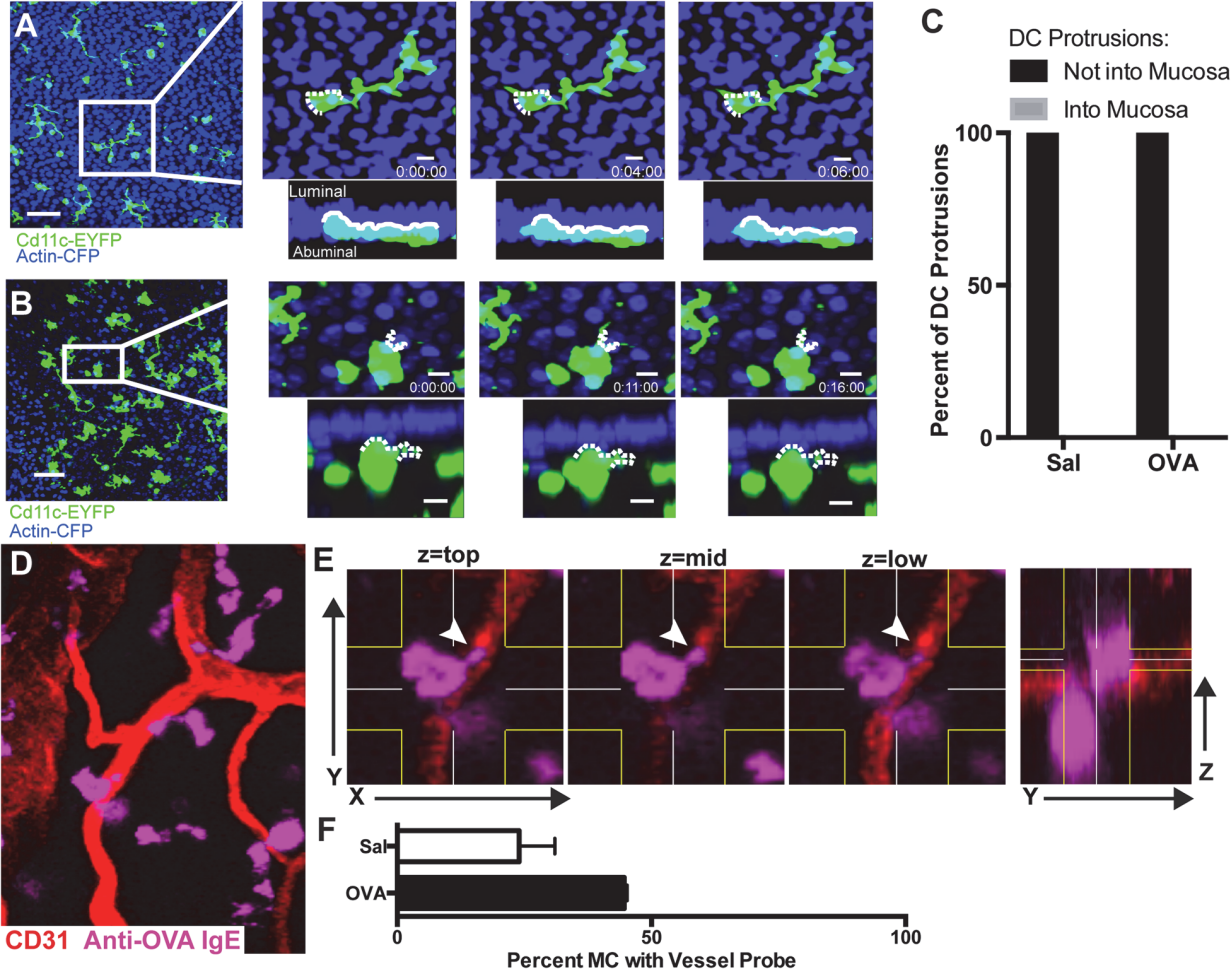
DC- and MC-probing in PBS- and OVA-challenged tracheas. (**A**) shows stills from a time lapse movie of a DC from a CD11c-EYFP x Actin-CFP mouse in a PBS-treated trachea and YZ rendering of the same data. Dendritic cell projections are shown moving over time, with a dotted white line marking the cell border at t = 0. See also **S3 Movie**. (**B**) contains still images from a second time lapse movie of a dendritic cell from a CD11c-EYFPxActin CFP mouse in an OVA-challenged trachea. Bars: XY = 50 μm; YZ = 10 μm. See also **S4 Movie**. (**C**) Protrusions of DC dendrites in 4 tracheas measured to show whether the projection entered the epithelium. (**D**) Still images from a time-lapse of a MC stained with anti-OVA IgE Alexa 647 (pink) near blood vessels stained by anti-CD31 PE (red). MCs protruding into vessel walls are marked by arrows. (**E**) Three XYZ-slices are shown, encompassing a thickness of 5 μm and separated from the next by 5 μm, illustrating that the tip of a MC lies near the vessel lumen. The rightmost panel shows a YZ rendering of the same dataset, illustrating the Z-volume rendered in mid-section. **(F)** The percentage of MC with protrusions that enter the vessel as defined by z-slices was quantified. * P = 0.007.

Finally, we sought to learn more about how DC and MC probe their environment under baseline conditions and after allergen challenge. In particular, previous reports had proposed that dendrites of tracheal DC should protrude across the tracheal epithelium [7]. We sought to assess this possibility, given that DC dendrites have been observed in lung alveoli. Analysis of hundreds of DC showed that they did indeed have very active dendrites that extended and retracted over minutes (**Fig. 4A-B** and **S3 Movie**). We observed these protrusions in DC making multiple (**Fig. 4A**) or single (**Fig. 4B**) projections but in all cases the DC were beneath the epithelium and did not have dendrites that projected into the tracheal lumen. Lumen-directed DC protrusions were not found in either control or allergen-bound conditions (**Fig. 4C**). Altogether, this feature most closely resembles that of cutaneous Langerhans cells, which have projections oriented laterally and parallel to layers of the epidermis [12].

In contrast, MC were found to have projections from the cell body, but these were less dynamic (see **S4 Movie**) and many appeared to project into blood vessels (**Fig. 4D**). This resembled MC projections in the skin, previously observed by Cheng et al. [3] and we confirmed that the tips of these projections lay within or extremely close to the lumen of the vessels (**Fig. 4E**). Notably, the frequency of these projections increased approximately 2-fold under allergen challenge.

## Discussion

Here, we identified the location and defined the motility modes of DC and MC, two cell types that are thought to be critical in allergen surveillance in airways. In the mouse trachea, most DC were in the mucosa and had cellular projections oriented perpendicular to the epithelial surface but not into the airway lumen. This feature is reminiscent of Langerhans cells in the skin. Most MC in this setting were well beneath the epithelium and had probing interactions with blood vessels, a behavior that was more frequent after allergic challenge, but not with the epithelium. The observation that neither DC nor MC projected processes into the airway lumen suggests that either transport of materials or barrier breach would be necessary to provide access to allergens. Towards the latter, the molecular sieving action of epithelial tight junctions could permit harmless self-antigens to cross, to educate and ‘tolerize’ the immune response to environmental antigens. In contrast, epithelial damage due to bacterial, viral, physical or other insults could result in material being presented to either MC or DC, in concert with pathogen-associated molecular patterns (PAMPs) or similar cues that encourage immune activation.

The vital preparation and labeling method we devised using the trachea represents a useful way to study tissue-localized immune responses in the airways. Although this study examined only basic features of these cells under steady state and allergic conditions, the preparation is a platform on which studies of the effects of viral infection or other pathological conditions could be undertaken. One of the most convincing pieces of evidence that the tracheal explant is a reasonable model is the observation of dendritic cells behaviors. Motility of DC dendrites is very sensitive to tissue viability and indeed is often considered a ‘canary in the coalmine’ for such the quality of a preparation [13]. Like the vital lung slice method, cellular viability is a feature that suggests that the trachea remained intact. Of course, for studies of processes that require lymphatic flow or blood flow, an intravital preparation would be required. Use of long projection (Gradient Index Refraction, GRIN) lenses could allow this to be achieved at high resolution.

By examining fixed specimens, we learned that most MC of the mouse lung are near the proximal main stem bronchus; few are in the lung periphery. The relative paucity and inaccessibility of MC in the lung encouraged us to use the trachea for further studies. Given the perceived importance of MC as histamine producers in lung allergic reactions, it was surprising to find so few of these cells in the lungs of C57BL/6 mice. One possible explanation for this is mouse-strain differences in MC density and localization. A second and related explanation is that MC share many features with basophils, such as the expression of Fc Epsilon receptors. In this study, we took advantage of recently defined mast-cell specific proteases and basophil-reporter strains to specifically exclude basophils from analysis. ‘Mast Cells’ identified in some previous reports, could turn out to be better named ‘basophils’. In that respect, basophils, as we defined them by the Basoph8 reporter, were abundant in OVA treated lungs [10]. Indeed as these lineages are unraveled, ‘mast cell’ histamine may be actually be found to come from basophils in some settings. A similar nomenclature issue concerns CD11c as a DC marker, and it may become more appropriate to refer to the tracheal DCs identified in our study as ‘macrophages’, as these lineages have many similarities. To that extent, some previous studies of airways also report that many DC/macrophages spread in the plane of the epithelial basement membrane rather than penetrating deeper into the mucosa [14].

## Materials and Methods

### Mice

All mice were bred and housed in specific pathogen–free housing and in strict accordance with the guidelines of the Laboratory Animal Resource Center of the University of California, San Francisco. All mice were C57BL/6 background. CD11c-EYFP [4] transgenic reporter mice were provided by M. Nussenzweig (The Rockefeller University, New York, NY), and Basoph8 × Rosa-DTα mice were provided by R. Locksley (University of California, San Francisco, San Francisco, California). CD11c-EYFP mice were crossed to Actin-CFP mice [15] obtained from I. Weissman (Stanford University, Stanford, CA). For all experiments, 6–8 week old mice were anesthetized using tribromoethanol or a mixture of ketamine and xylazine. Approval for the use of mice in this study was obtained from the Institutional Animal Care and Use Committee of the University of California, San Francisco.

### OVA sensitization and challenge protocol

We used a murine model of asthma based on administration of OVA and adjuvant [16]. In brief, endotoxin-depleted ovalbumin (Sigma) [17] (0.25 mg/ml in alum (Sigma), 5 mg/ml) in a total volume of 200 μl was injected intraperitoneally on days 0, 7, and 14. Mice were challenged intranasally with OVA (100 μg in 40 μl of phosphate buffered saline, PBS) or PBS alone on days 21, 22, and 23.

### Antibodies and reagents

Anti-OVA IgE clone 2C6 [9] was made in the UCSF Monoclonal Antibody Core. The antibody was then conjugated using a Life Technologies Alexa Fluor 647 Antibody Labeling Kit. Additional antibodies included R&D AF3767 goat anti-MMCP6 and Jackson ImmunoResearch Alexa 488 donkey anti-goat IgG 705-165-147. Dextrans, 10,000 MW (Life Technologies) labeled with Texas Red or Alexa 647, were also used.

### Fixed tissue staining

Anesthetized mice were perfused via the aorta with fixative (1% paraformaldehyde (PFA) in PBS (pH 7.4) for 2 minutes. Tracheas and lungs were removed from the chest and immersed in fixative for 1 hour at room temperature, then washed multiple times with PBS. Some tracheas and all lungs were immersed overnight in 30% sucrose solution, embedded the next day in OCT medium (Sakura Finetek), frozen, and sections were cut in a cryostat at a thickness of 100 to 200 μm. For immunohistochemistry, tissues were stained overnight at room temperature as whole mounts or thick sections immersed in primary antibodies in PBS containing 0.3% Triton X-100 + 0.1% sodium azide (as an anti-bacterial agent) + 0.2% bovine serum albumin + 5% normal donkey serum (as a blocking agent). Primary antibodies included anti-mMCP6, used at a dilution of 1:500, and anti-OVA Alexa 647-IgE (UCSF), used at 1:200. After staining with primary antibodies, tissues were washed multiple times with PBS + 0.3% Triton, incubated overnight with secondary antibodies, then fixed for 10 minutes in 1% PFA and mounted using Vectashield (Vector Laboratories). Specimens were imaged with a Zeiss 510 confocal microscope (Thornwood, NY) using Zeiss Aim software version 4.0.

### Live imaging of tracheas after antibody staining

Mice were given a lethal dose of tribromoethanol and exsanguinated from the descending aorta before dissection. The trachea and bronchi were cut along the ventral midline while still attached to the larynx and lungs. The trachea was then removed and placed into RPMI medium and stained using Anti-OVA IgE Alexa 647 (UCSF) at a concentration of 5 μg/ml in 200 μl of RPMI for one hour, with or without anti-CD31-PE at 1 μg/ml. Tracheas were then washed every 10 minutes for one hour in RPMI. After washing, tracheas were pinned at the four corners using insect pins in Sylgard-based Petri dish filled with RPMI and were maintained at 37°C until imaging. During imaging, a superfusion system kept RPMI (at 37°C and bubbled with a carbon dioxide/oxygen mixture) flowing over the tracheas.

### Confocal and real-time 2-photon imaging

A custom resonant-scanning 2-photon instrument [18] that has a four-photomultiplier tube detector and collects data at video rate was used for multiphoton imaging. Samples were excited with a 10-W MaiTai Ti-Sapphire laser (Spectra-Physics) tuned to a wavelength of 910 nm, and emission wavelengths of 440/440 nm (for Hoechst and CFP), 505/520 nm (for GFP), 542/527nm (for YFP), and 605/670 nm (for Alexa 647) were collected. For some experiments, we alternatively used a Nikon A1R multiphoton/ confocal microscope equipped with a MaiTai DeepSee laser. Each lung section was first surveyed in a raster scan spanning 1567 μm x 1300 μm x 175 μm in XYZ axes. For time-lapse image acquisition, each XY stack spanned 313 μm x 260 μm at a resolution of 0.653 μm per pixel spaced 1 μm apart for approximately 100 μm in the Z axis, and 10–20 video-rate frames were averaged. Images were analyzed using Imaris (Bitplane), Fiji (Open Source), and Matlab (Math Works) software.

## Supporting Information

S1 Movie 3D rotation and time-lapse movie of MC and DC in a region of living trachea 1 day after challenge with OVA allergen followed by time-lapse of cell movement within this region. MC (pink) are relatively sessile, as are DC (green), but DC have extensions/retractions of dendrites. See also Fig. 3.
(MOV)

S2 Movie 3D rotation of a contact between MC and DC. MC (red) in the layer adjacent to DC (green) make close contact. Only a fraction of MC have such contacts, and this fraction is similar with and without allergen challenge (see also Fig. 3).
(MOV)

S3 Movie Dendrites forming dynamic projections in the plane of the tracheal epithelium. DC (CD11cYFP, shown as green) are shown in a trachea in which nuclei of epithelial cells were labeled blue by transient immersion in Hoechst 33342.
(MOV)

S4 Movie Three projections of the dynamics of MC during probing of a blood vessel. MC stained with anti-OVA IgE Alexa 647 (pink) along with staining for blood vessels using anti-CD31 PE (red). Data correspond to Fig. 4.
(MOV)
